# A genomic timescale of prokaryote evolution: insights into the origin of methanogenesis, phototrophy, and the colonization of land

**DOI:** 10.1186/1471-2148-4-44

**Published:** 2004-11-09

**Authors:** Fabia U Battistuzzi, Andreia Feijao, S Blair Hedges

**Affiliations:** 1 NASA Astrobiology Institute and Department of Biology, 208 Mueller Laboratory, The Pennsylvania State University, University Park, PA 16802, USA; 2 European Molecular Biology Laboratory, Meyerhofstrasse 1, 69117 Heidelberg, Germany

## Abstract

**Background:**

The timescale of prokaryote evolution has been difficult to reconstruct because of a limited fossil record and complexities associated with molecular clocks and deep divergences. However, the relatively large number of genome sequences currently available has provided a better opportunity to control for potential biases such as horizontal gene transfer and rate differences among lineages. We assembled a data set of sequences from 32 proteins (~7600 amino acids) common to 72 species and estimated phylogenetic relationships and divergence times with a local clock method.

**Results:**

Our phylogenetic results support most of the currently recognized higher-level groupings of prokaryotes. Of particular interest is a well-supported group of three major lineages of eubacteria (Actinobacteria, *Deinococcus*, and Cyanobacteria) that we call Terrabacteria and associate with an early colonization of land. Divergence time estimates for the major groups of eubacteria are between 2.5–3.2 billion years ago (Ga) while those for archaebacteria are mostly between 3.1–4.1 Ga. The time estimates suggest a Hadean origin of life (prior to 4.1 Ga), an early origin of methanogenesis (3.8–4.1 Ga), an origin of anaerobic methanotrophy after 3.1 Ga, an origin of phototrophy prior to 3.2 Ga, an early colonization of land 2.8–3.1 Ga, and an origin of aerobic methanotrophy 2.5–2.8 Ga.

**Conclusions:**

Our early time estimates for methanogenesis support the consideration of methane, in addition to carbon dioxide, as a greenhouse gas responsible for the early warming of the Earths' surface. Our divergence times for the origin of anaerobic methanotrophy are compatible with highly depleted carbon isotopic values found in rocks dated 2.8–2.6 Ga. An early origin of phototrophy is consistent with the earliest bacterial mats and structures identified as stromatolites, but a 2.6 Ga origin of cyanobacteria suggests that those Archean structures, if biologically produced, were made by anoxygenic photosynthesizers. The resistance to desiccation of Terrabacteria and their elaboration of photoprotective compounds suggests that the common ancestor of this group inhabited land. If true, then oxygenic photosynthesis may owe its origin to terrestrial adaptations.

## Background

The evolutionary history of prokaryotes includes both horizontal and vertical inheritance of genes [1-3]. Horizontal gene transfer (HGT) events are of great interest in themselves, for their roles in creating functionally new combinations of genes [4], but they pose problems for investigating the phylogenetic history and divergence times of organisms. The existence of a core of genes that has not been transferred is still under debate as HGTs have been detected in genes previously considered to be immune to these events [2,5-11]. Although a complete absence of HGT appears to be unlikely, genes belonging to different functional categories seem to be horizontally transferred with different frequencies [11-13]. Genes forming complex interactions with other cellular components (e.g. translational proteins) have a lower frequency of HGT and are generally more conserved among organisms. Recent studies based on analyses of these genes have obtained similar phylogenies suggesting an underlying phylogenetic signal [3,14-17]. If we accept the use of core genes for phylogeny reconstruction then they should also be of use for time estimation with molecular clocks. Moreover the increasing number of prokaryotic genomes available has facilitated the detection of HGT through more accurate detection of orthology, paralogy, and monophyletic groups, and the concatenation of gene and protein sequences has helped increase the confidence of nodes and decrease the variance of time estimates [14,16,18,19].

Temporal information concerning prokaryote evolution has come from diverse sources. For eukaryotes, the fossil record provides an abundant source of such data, but this has not been true for prokaryotes, which are difficult to identify as fossils [20,21]. Limited information on specific groups or metabolites has been obtained from analyses of isotopic concentrations [22] and detection of biomarkers [23,24]. By making some simple assumptions – e.g., that aerobic organisms evolved after oxygen became available [25]- it is possible to constrain some nodes in the prokaryote timescale, but only in a coarse sense. However, most information on the timescale of prokaryote evolution has come from analysis of DNA and amino acid sequence data with molecular clocks [26-30]. The detection of evolutionary patterns in metabolic innovations, as a consequence of a phylogeny not dominated by HGT events, allows more detailed constraints on a prokaryote timescale.

In contrast to conventional interpretations of cyanobacteria as being among the most ancient of life forms on Earth [31], these studies have consistently found a late origin of cyanobacteria [28,30], nearly contemporaneous with the major Proterozoic rise in oxygen at 2.3 billion years ago (Ga), termed the Great Oxidation Event (GOE) [32].

In this study we have assembled a data set of amino acid sequences from 32 proteins common to 72 species of prokaryotes and eukaryotes and estimated phylogenetic relationships and divergence times with a local clock method. These results in turn have been used to investigate the origin of metabolic pathways of importance in evolution of the biosphere.

## Results

### Data set

The majority (81%) of the 32 proteins that were used are classified in the "information storage and processes" functional category of the COG. The other categories represented are "cellular processes" (10%), "metabolism" (3%), and "information storage and processing" + "metabolism" (proteins with combined functions; 6%). Other studies that have analyzed prokaryote genome sequence data for phylogeny have found a similar high proportion of proteins in the "information storage and processes" functional category, presumably because HGT is more difficult with such genes that are vital for the survival of the cell [3,18,33,34].

The concatenated and aligned data set of 32 proteins contained 27,205 amino acid sites (including insertions and deletions). With alignment gaps removed, the two data sets analyzed were 7,338 amino acid sites (Archaebacteria) and 7,597 amino acid sites (Eubacteria). The data sets were complete in the sense that sequences of all taxa were present for all proteins.

### Phylogeny

**Figure 1:**
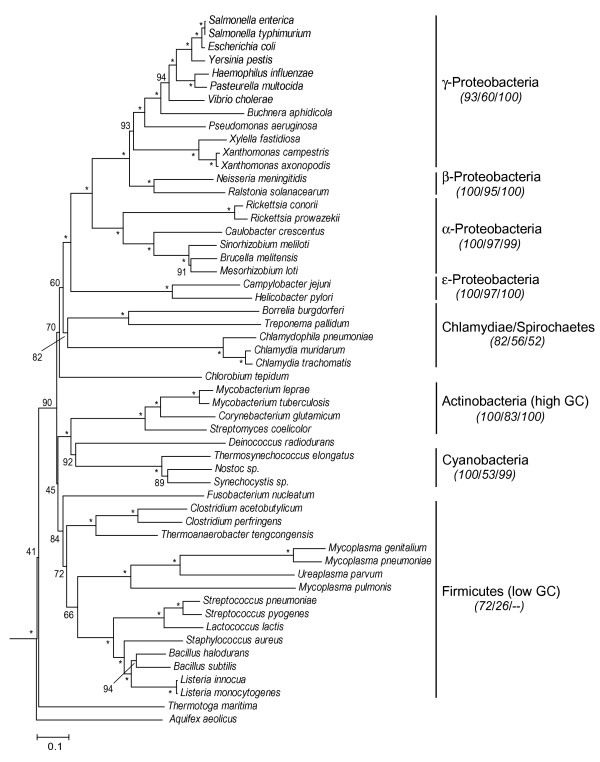
Phylogenetic tree (ME; α = 0.94) of eubacteria rooted with archaebacteria, using sequences of 32 proteins (7,597 amino acids). Bootstrap values are shown on nodes; asterisks indicate support values > 95%. For major groups, support values from three phylogenetic methods (ME/ML/Bayesian) are indicated in italics (dash indicates a group was not present).

The phylogeny of eubacteria (Fig. 1) shows significant bootstrap support for most of the major groups and subgroups. All proteobacteria form a monophyletic group (support values 95/47/99 for ME, ML and Bayesian respectively) with the following relationships of the subgroups: (epsilon (alpha (beta, gamma))). There has been debate about the effect of base composition and substitution rate on the phylogenetic position of the endosymbiont *Buchnera *among γ-proteobacteria [35,36]. Its position here (Fig. 1) differs slightly from both studies; accordingly, any conclusions concerning its divergence time should be treated with caution. Spirochaetes cluster with Chlamydiae, Actinobacteria with Cyanobacteria and *Deinococcus *(support values for Cyanobacteria + *Deinococcus *are 92/80/99) and the hyperthermophiles (*Thermotoga*, *Aquifex*) branch basally in the tree. These groups and relationships are similar to those found previously with analyses of prokaryote genome sequences [3,18,33,34].

**Figure 2:**
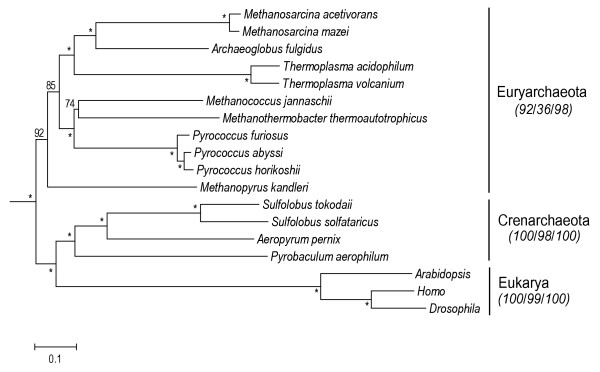
Phylogenetic tree (ME; α = 1.20) of archaebacteria rooted with eubacteria, using sequences of 32 proteins (7,338 amino acids). Bootstrap values are shown on nodes; asterisks indicate support values > 95%. For major groups, support values from three phylogenetic methods (ME/ML/Bayesian) are indicated in italics.

The phylogeny of archaebacteria (Fig. 2) agrees with some but not all aspects of previous phylogenetic analyses of prokaryote genomes using sequence data [3,14,18,30,37,38] and the presence and absence of genes [37,39-41]. For example, each of the two major clades of Archaebacteria (excluding Korarchaeota, which was not represented) is monophyletic. This is consistent with some analyses [14,18] but not others [3]. Also, the position of Crenarchaeota as closest relatives of eukaryotes (Fig. 2), instead of Euryarchaeota, has been debated [14,18,30,42,43]. The faster rate of evolution in eukaryotes (Fig. 2), as noted elsewhere [30,44], requires some caution in drawing conclusions regarding their phylogenetic position. Methanogens were found to be monophyletic in some previous analyses [3,41] but were paraphyletic in other analyses [38,45,46] and in our analysis (Fig. 2). The phylogenetic position of one species of methanogen in particular, *Methanopyrus kandleri*, has differed among previous studies [47-49]. However, it is difficult to make direct comparisons among various studies because they have included different sets of taxa.

### Time estimation

Times of divergence were estimated for all nodes in the phylogenies of eubacteria (Fig. 1) and archaebacteria (Fig. 2) using the alternative constraints (calibrations) described in the Methods. The eubacteria time estimates show an average 7% increase from the molecular to the geologic (2.3 Ga minimum) calibration point. Two other additional geologic calibration points were used in the analyses (see Methods), 2.3 Ga fixed and 2.7 Ga minimum, which showed respectively 10% younger and 11% older time estimates compared with the 2.3 Ga minimum calibration point.

The times estimated with the fossil calibration point in the archaebacteria data set were on average only 10% younger than the ones estimated with the molecular calibration. Moreover there was even a smaller effect on the time estimates of the deepest nodes, which were the ones of interest in this study (node M 3.2%, node N 2.1%, node O 1.8% and node P 1.3%). This variation is due not only to the different calibration times but also to the type of constraints used (i.e. minimum boundaries only vs. minimum and maximum bounds).

**Figure 3:**
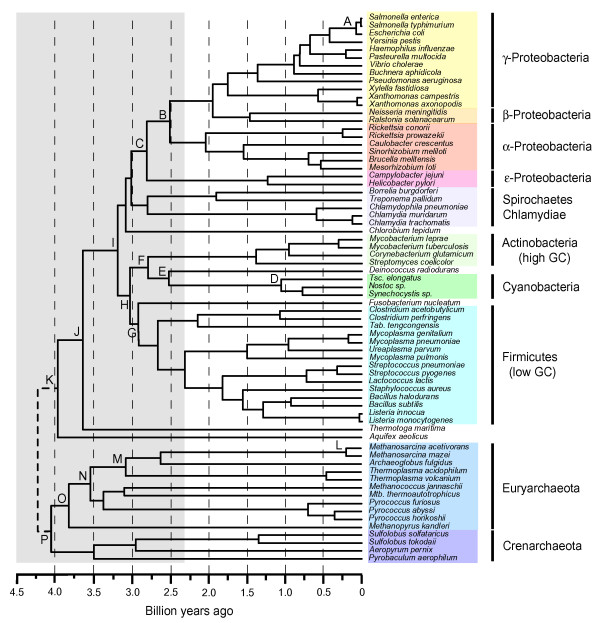
A timescale of prokaryote evolution. Letters indicate nodes discussed in the text. The last common ancestor was arbitrarily placed at 4.25 Ga in the tree, although this placement was not part of the analyses. The grey rectangle shows the time prior to the initial rise in oxygen (presumably anaerobic conditions). Mtb: *Methanothermobacter*, Tab: *Thermoanaerobacter*, Tsc: *Thermosynechococcus*.

A single timetree (Fig. 3) was constructed from the phylogenetic and divergence time data. The time estimates summarized in that tree derive only from the best-justified calibrations. For eubacteria, the 2.3 Ga minimum calibration (constraint), from the geologic record, was chosen because it encompasses all of the hypothesized time estimates for the origin of cyanobacteria. For archaebacteria, the 1.2 Ga calibration (minimum 1.174 Ga, maximum 1.222 Ga), from the red algae fossil record, was selected because it provides a conservative constraint on the divergence of plants and animals. Time estimates and 95% credibility intervals for all nodes under all calibrations are presented elsewhere [see Additional file 1, Additional file 2, and Additional file 3], and those data are summarized for selected nodes and calibrations for eubacteria and archaebacteria (Table 1). Although some undetected HGT could be a source of bias in the time estimates, the direction of the bias (raising or lowering the estimate) would depend on the specific node and groups involved, and it is unlikely to have had a major affect on the results, even if present.

**Table 1:** Time estimates for selected nodes in the tree of eubacteria (A-K) and archaebacteria (L-P). Letters refer to Fig. 3.

	**Time (Ma)**^a^	**CI**^b^
**Node A**	102	57–176
**Node B**	2508	2154–2928
**Node C**	2800	2452–3223
**Node D**	1039	702–1408
**Node E**	2558	2310–2969
**Node F**	2784	2490–3203
**Node G**	2923	2587–3352
**Node H**	3054	2697–3490
**Node I**	3186	2801–3634
**Node J**	3644	3172–4130
**Node K**	3977	3434–4464
**Node L**	233	118–386
**Node M**	3085	2469–3514
**Node N**	3566	2876–3948
**Node O**	3781	3047–4163
**Node P**	4112	3314–4486

^a ^Averages of the divergence times estimated using the 2.3 Ga minimum constraint and the five ingroup root constraints (nodes A-K) and using the 1.198 ± 0.022 Ga constraint and the five ingroup root constraints (nodes L-P).

^b ^Credibility interval (minimum and maximum averages of the analyses under the five ingroup root constraints)

Divergence times within eubacteria (Fig. 3, Table 1, nodes A-K) show a pattern seen previously [30] whereby most major groups diverge from one another (nodes B-I excluding node D) in a relatively limited time interval, approximately between 2.5–3.2 Ga. The position of the hyperthermophiles has been debated, with some studies showing them in a basal position whereas others place them more derived. The high G-C composition of these taxa is believed to be responsible for this difficulty in phylogenetic placement. Here, they branch basally (node J, 3.17–4.13 Ga and node K, 3.43–4.46 Ga), but this should be interpreted with caution for this reason. The divergence of *Escherichia coli *from *Salmonella typhimurium *(Fig. 3, Table 1, node A; 0.06–0.18 Ga) is consistent with the time estimated previously from consideration of mammalian host evolution (0.12–0.16 Ga) [26]. On the other hand an inconsistency with the fossil record is represented by the divergence of unicellular (*Thermosynechococcus elongatus*) and heterocyst-forming (*Nostoc sp.*) cyanobacteria. Our time estimate for this divergence is 0.70–1.41 Ga (Fig. 3, Table 1, node D) while microfossils of both groups have been identified in Mesoproterozoic (1.5–1.3 Ga) and Paleoproterozoic (2.12–2.02 Ga) rocks [50-52]. However the identification of these latter fossils has been debated [51]. Branch lengths of cyanobacteria in our protein tree and in 16S ribosomal RNA trees [34] do not suggest obvious substitutional biases or rate changes as they are neither unusually long nor unusually short. The reason for the discrepancy between the molecular and fossil times remains unclear but a possible misinterpretation of the fossil record cannot be dismissed.

Divergence times of most internal nodes among archaebacteria (Fig. 3, Table 1, nodes L-P) are closely spaced in time and relatively ancient, approximately between 3.1–4.1 Ga, regardless of the initial setting (prior) for the ingroup root. Node P is the earliest divergence, separating Euryarchaeota from Crenarchaeota+eukaryotes. Node O represents the common ancestor of the methanogens in our analysis (*Methanopyrus kandleri*, *Methanothermobacter thermoautotrophicus*, *Methanococcus jannaschii*, *Archaeoglobus fulgidus*, *Methanosarcina mazei *and *M. acetivorans*). Therefore, methanogenesis presumably arose between nodes P and O, or between 4.11 Ga (3.31–4.49 Ga) and 3.78 Ga (3.05–4.16 Ga) (Fig. 3, Table 1). If the position of *Methanopyrus kandleri *is not considered, in lieu of the current debate concerning its relationships (noted above), node N (Fig. 3, Table 1), the minimum time for the origin of methanogenesis drops only slightly, from 3.78 Ga (3.05–4.16 Ga) to 3.57 Ga (2.88–3.95 Ga).

## Discussion

### Origin of life on Earth

Neither the time for the origin of life, nor the divergence of archaebacteria and eubacteria, was estimated directly in this study. Nonetheless, one divergence within archaebacteria was estimated to be as old as 4.11 Ga (Node P), suggesting even earlier dates for the last common ancestor of living organisms and the origin of life. This is in agreement with previous molecular clock analyses using mostly different data sets and methodology [28,30]. A Hadean (4.5–4.0 Ga) origin for life on Earth is also consistent with the early establishment of a hydrosphere [31,53]. Nevertheless, the earliest geologic and fossil evidence for life has been debated [21,54-59] leaving no direct support for such old time estimates.

### Methanogenesis

The lower luminosity of the sun during the Hadean and Archean predicts that surface water would have been frozen during that time. Instead there is evidence of liquid water and moderate to high surface temperatures [60,61]. The long term carbon cycle (carbonate-silicate cycle), which acts as a temperature buffer, combined with greenhouse gases, probably explain this "Faint Young Sun Paradox" [61]. Arguments have been made in support of either methane [62-64] or carbon dioxide [65] as the major greenhouse gas involved. If methane was important, it would have necessarily come from organisms (methanogens), given the volume required.

Archaebacteria are the only prokaryotes known to produce methane. Our time estimate of between 4.11 Ga (3.31–4.49 Ga) and 3.78 Ga (3.05–4.16 Ga) for the origin of methanogenesis suggests that methanogens were present on Earth during the Archean, consistent with the methane greenhouse theory [64]. Nonetheless, this does not rule out the alternative (carbon dioxide) explanation [65].

### Anaerobic methanotrophy

Anaerobic methanotrophy, or anaerobic oxidation of methane (AOM), is a metabolism associated with anoxic marine sediments rich in methane. This metabolism is characterized by the coupling of two reactions, oxidation of methane and sulfate reduction. The methane oxidizers are represented by archaebacteria phylogenetically related to the Methanosarcinales, while the sulfate reducers, when present, are eubacterial members of the δ-proteobacteria division [66]. These two groups of prokaryotes have been found associated in syntrophies, thus suggesting the coupling of these two pathways [66-69]. Archaebacteria have been found also isolated in monospecific clusters, oxidizing methane through an unknown reaction. It has been suggested that they may use elements of both the methanogenesis and sulfate-reducing pathways [70]. An example of coexistence of genes from both of these pathways is *Archaeoglobus fulgidus*. The particular condition of this archaebacterium has been explained with an ancient horizontal gene transfer from an eubacterial lineage, most likely a δ-proteobacterium [71,72].

The phylogenetic position of the anaerobic methanotrophs with the Methanosarcinales places the maximum date for the origin of this metabolism at 3.09 (2.47–3.51) Ga (Fig. 3, Table 1, node M). The minimum time estimate of 0.23 Ga (0.12–0.39 Ga) (Fig. 3, Table 1, node L), probably a substantial underestimate of the true time, results from the limited phylogenetic sampling available for this group.

### Aerobic methanotrophy

Aerobic methanotrophs are represented in the α and γ divisions of the proteobacteria. This suggests an origin for this metabolism between node C (2.80 Ga; 2.45–3.22 Ga) and node B (2.51 Ga; 2.15–2.93 Ga) (Fig. 3, Table 1). Shared genes from this pathway and from methanogenesis also have been found in the Planctomycetales [73]. This has suggested a revision of the direction of the HGT, usually considered from archaebacteria to eubacteria [1], that presumably has spread these genes in the two domains. However the absence of Planctomycetales from our dataset and its controversial phylogenetic position [74] does not allow us to discriminate among these possibilities.

Both anaerobic and aerobic methanotrophy have been used to explain the highly depleted carbon isotopic values found in 2.8–2.6 Ga geologic formations [22,75]. Our time estimates for these two metabolisms are both compatible with the isotopic record. Molecular clock methods have estimated the origin of cyanobacteria at 2.56 Ga (2.04–3.08 Ga) [30]. Because oxygenic photosynthesis would have been necessary for aerobic methanotrophy [75], an anaerobic metabolism seems more likely to explain the isotopic record.

### Phototrophy

The ability to utilize light as an energy source (phototrophy, photosynthesis) is restricted to eubacteria among prokaryotes. Phototrophic eubacteria are found in five major phyla (groups), including proteobacteria, green sulfur bacteria, green filamentous bacteria, gram positive heliobacteria, and cyanobacteria [4,76]. Only cyanobacteria produce oxygen.

There are three explanations for this broad taxonomic distribution of phototrophic metabolism; it evolved in one lineage of eubacteria and spread at a later time to other lineages by horizontal transfer, the common ancestor of these groups possessed this metabolism and genetic machinery, or there was a combination of horizontal transfer and vertical inheritance [4]. Because two of the three explanations require a phototrophic common ancestor, and because some features of the Archean geologic record require this metabolism if biologically produced [77], we have assumed here that the common ancestor (Node I) was phototrophic.

Therefore, we estimate that phototrophy evolved prior to 3.19 (2.80–3.63) Ga (Fig. 3, Table 1, node I). Because the hyperthermophiles *Aquifex *and *Thermotoga *are not phototrophic and branch more basally, 3.64 (3.17–4.13) Ga (Node J) can be considered a maximum date for phototrophy. However, if those hyperthermophiles instead occupy a more derived position on the tree, as some analyses have indicated [33], then the maximum date is no longer constrained in this analysis.

### The colonization of land

The evolution of phototrophy was most likely linked to the evolution of other features essential to survival in stressful environments. Considerable biological damage can occur from exposure to ultraviolet radiation, especially prior to the GOE and later formation of the protective ozone layer [78]. The synthesis of pigments such as carotenoids, which function as photoprotective compounds against the reactive oxygen species created by UV radiation [79], is an ability present in all the photosynthetic eubacteria and in groups that are partly or mostly associated with terrestrial habitats such as the actinobacteria, cyanobacteria, and *Deinococcus*-*Thermus*.

Pigmentation was probably a fundamental step in the colonization of surface environments [80]. Besides the sharing of photoprotective compounds, these three groups (cyanobacteria, actinobacteria, and *Deinococcus*) also share a high resistance to dehydration [81-84], which further suggests that their common ancestor was adapted to land environments. Therefore we propose the name Terrabacteria (L. *terra*, land or earth) for the group that includes the bacterial phyla Actinobacteria, Cyanobacteria, and *Deinococcus-Thermus*. An early colonization of land is inferred to have occurred after the divergence of this terrestrial lineage with Firmicutes (Fig. 3, Table 1, node H), 3.05 (2.70–3.49) Ga, and prior to the divergence of Actinobacteria with Cyanobacteria + *Deinococcus *(Fig. 3, Table 1, node F), 2.78 (2.49–3.20) Ga. These molecular time estimates are compatible with time estimates (2.6–2.7 Ga) based on geological evidence for the earliest colonization of land by organisms (prokaryotes) [85]. Many groups of prokaryotes currently inhabit terrestrial environments, indicating that land has been colonized multiple times in different lineages.

### Oxygenic photosynthesis

From the above analyses and discussion, some of the early steps leading to oxygenic photosynthesis apparently were acquisition of protective pigments, phototrophy, and the colonization of land. Currently, hundreds of terrestrial species of cyanobacteria are known, broadly distributed among the orders, with species occurring in some of the driest environments on Earth. It is possible that a terrestrial ancestry of cyanobacteria, where stresses resulting from desiccation and solar radiation were severe, may have played a part in the evolution of oxygenic photosynthesis. Nonetheless, there is ample evidence that horizontal gene transfer also has played an important role in the assembly of photosynthetic machinery [4].

Although we have used the origin of cyanobacteria as a calibration (2.3 Ga, geologic time based on GOE), such minimum constraints permit the estimated time to be much older in a Bayesian analysis. However, in this case, the time estimated for node E (2.56 Ga; 2.31–2.97 Ga; Fig. 3, Table 1) was not much older than the constraint itself. It also agrees with an earlier molecular time estimate (2.56 Ga; 2.04–3.08 Ga) based on a largely different data set and methods [30]. When we used the older minimum constraint of 2.7 Ga, corresponding to 2α-methyl-hopane evidence considered to represent a biomarker of cyanobacteria [86], the estimated time was likewise only slightly older [see Additional file 1]. The oldest time estimates for oxygenic photosynthesis that we obtained are still considerable younger than has been assumed – generally – in the geologic literature [31,32,87]. This suggests that carbon isotope excursions, microfossils, microbial mats, stromatolites, and other pre-3 Ga evidence ascribed to cyanobacteria should be re-evaluated.

## Conclusions

**Figure 4:**
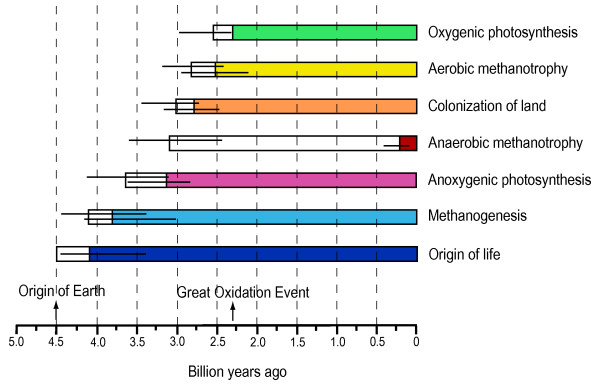
A time line of metabolic innovations and events on Earth. The minimum time for oxygenic photosynthesis is constrained by the Great Oxidation Event (2.3 Ga) whereas the maximum time for the origin of life is constrained by the origin of Earth (4.5 Ga). Horizontal lines indicate credibility intervals, white boxes indicate minimum and maximum time constraints on the origin of a metabolism or event, and colored boxes indicate the presence of the metabolism or event.

The analyses presented here are based on the assumption, still under debate, that historical information (phylogenies and divergence times) can be retrieved from genes in the prokaryote genome that have not been affected by horizontal gene transfer. Our prokaryotic timeline shows deep divergences within both the eubacterial and archaebacterial domains indicating a long evolutionary history. The early evolution of life (>4.1 Ga) and early origin of several important metabolic pathways (phototrophy, methanogenesis; but not oxygenic photosynthesis) suggests that organisms have influenced the Earth's environment since early in the history of the planet (Fig. 4). An inferred early presence of methanogens (3.8–4.1 Ga) is consistent with models suggesting that methane was important in keeping the Earth's surface warm in the Archean but does not rule out the possibility that carbon dioxide may have been equally (or more) important. In contrast to many classical interpretations of the early evolution of life, we find no compelling evidence for a pre-3 Ga evolution of cyanobacteria and oxygenic photosynthesis. This unique metabolism apparently evolved relatively late in the radiation of eubacterial clades, shortly before the Great Oxidation event (~2.3 Ga). The evolution of oxygenic photosynthesis may have involved a combination of adaptations to stressful terrestrial environments as well as acquisition of genes through horizontal transfer.

## Methods

### Data assembly

We assembled a dataset that maximized the number of taxa and proteins from available organisms with complete genome sequences of prokaryotes and selected eukaryotes. In doing so, we omitted a few taxa (e.g., *Agrobacterium tumefaciens Cereon str C58 *and *Halobacterium sp. NRC-1*) whose addition to the data set would have resulted in a substantial reduction in the total number of proteins. Data assembly began with the Clusters of Orthologous Groups of Proteins (COG) [88], which consisted of 84 proteins common to 43 species. With that initial dataset we added other species from among completed microbial genomes (NCBI; National Center for Biotechnology Information), assisted by BLAST and PSI-BLAST [89]. In total 72 species were included in the study (54 eubacteria, 15 archaebacteria and three eukaryotes).

The species of Archaebacteria and their accession numbers are: *Aeropyrum pernix K1 *(NC_000854), *Archaeoglobus fulgidus *(NC_000917), *Methanothermobacter thermoautotrophicus str. Delta H *(NC_000916), *Methanococcus jannaschii *(NC_000909), *Methanopyrus kandleri AV19 *(NC_003551), *Methanosarcina acetivorans str. C2A *(NC_003552), *Methanosarcina mazei Goe1 *(NC_003901), *Pyrobaculum aerophilum *(NC_003364), *Pyrococcus abyssi (*NC_000868), *Pyrococcus furiosus DSM 3638 *(NC_003413), *Pyrococcus horikoshii *(NC_000961), *Sulfolobus solfataricus *(NC_002754), *Sulfolobus tokodaii *(NC_003106), *Thermoplasma acidophilum *(NC_002578), *Thermoplasma volcanium *(NC_002689).

The species of Eubacteria are: *Aquifex aeolicus *(NC_000918), *Bacilllus halodurans *(NC_002570), *Bacillus subtilis *(NC_000964), *Borrelia burgodorferi *(NC_001318), *Brucella melitensis *(NC_003317, NC_003318), *Buchnera aphidicola str. APS (Acyrthosiphon pisum) *(NC_002528), *Campylobacter jejuni *(NC_002163), *Caulobacter crescentus CB15 *(NC_002696), *Chlamydia muridarum *(NC_002620), *Chlamydia trachomatis *(NC_000117), *Chlamydophila pneumoniae CWL029 (*NC_000922), *Chlorobium tepidum str. TLS *(NC_002932), *Clostridium acetobutylicum *(NC_003030), *Clostridium perfringens *(NC_003366), *Corynebacterium glutamicum ATCC 13032 *(NC_003450), *Deinococcus radiodurans *(NC_001263, NC_001264), *Escherichia coli O157:H7 EDL933 *(NC_002655), *Fusobacterium nucleatum subsp. nucleatum ATCC 25586 *(NC_003454)*, Haemophilus influenzae Rd *(NC_000907), *Helicobacter pylori 26695 *(NC_000915), *Lactococcus lactis subsp. lactis *(NC_002662), *Listeria innocua *(NC_003212), *Listeria monocytogenes EGD-e *(NC_003210)*, Mesorhizobium loti *(NC_002678), *Mycobacterium leprae *(NC_002677), *Mycobacterium tuberculosis H37Rv *(NC_000962), *Mycoplasma genitalium G-37 *(NC_000908), *Mycoplasma pneumoniae *(NC_000912), *Mycoplasma pulmonis *(NC_002771), *Neisseria meningitidis MC58 *(NC_003112), *Nostoc sp. PCC7120 *(NC_003272), *Pasteurella multocida *(NC_002663), *Pseudomonas aeruginosa PA01 *(NC_002516), *Ralstonia solanacearum *(NC_003295), *Rickettsia conorii *(NC_003103), *Rickettsia prowazekii *(NC_000963), *Salmonella enterica subsp. enterica serovar Typhi *(NC_003198), *Salmonella typhimurium LT2 *(NC_003197), *Sinorhizobium meliloti *(NC_003047), *Staphylococcus aureus Mu50 *(NC_002758), *Streptococcus pneumoniae TIGR4 *(NC_003028), *Streptococcus pyogenes M1 GAS *(NC_002737), *Streptomyces coelicolor A3(2) *(NC_003888), *Synechocystis PCC6803 *(NC_000911), *Thermoanaerobacter tengcongensis *(NC_003869), *Thermosynechococcus elongatus BP-1 *(NC_004113), *Thermotoga maritima *(NC_000853), *Treponema pallidum subsp. pallidum str. Nichols *(NC_000919), *Ureaplasma parvum serovar 3 str. ATCC 700970 *(NC_002162), *Vibrio cholerae O1 biovar eltor str. N16961 *(NC_002505, NC_002506), *Xanthomonas campestris pv. campestris str. ATCC 33913 *(NC_003902), *Xanthomonas axonopodis pv. citri str. 306 *(NC_003919), *Xylella fastidiosa 9a5c *(NC_002488)*, Yersinia pestis *(NC_003143).

The eukaryotes were *Arabidopsis thaliana*, *Drosophila melanogaster*, *Homo sapiens*. Accession numbers for eukaryote proteins are presented elsewhere [90].

This dataset consisted of 60 proteins that were individually analysed as a step in orthology determination. The proteins were aligned with CLUSTALW [91]. Then phylogenetic trees of each protein were built and visually inspected. Initial trees were constructed using Minimum Evolution (ME), with MEGA version 2.1 [92]. The major criterion that we used in determining which genes to include or exclude was the monophyly of domains. We rejected genes with domains (archaebacteria and eubacteria) that were non-monophyletic, as these would be the best examples of HGT; this amounted to 61% of the genes rejected. Some other genes were omitted if there were detectable cases of HGT within a domain, such as the deep nesting of a species from one Phylum within a clade of another Phylum. Otherwise we did not eliminate genes that had a different branching order of phyla within a domain or different relationships of groups of lower taxonomic categories. Admittedly, ancient cases of HGT might be an explanation for some of those topological differences, but they are not detectable. However, we further tested the effectiveness of our criteria by examining the stability of individual protein trees, using different gamma values (α = 1, 0.5 and 0.3). We kept only the genes that were stable to such perturbations (in terms of remaining in that category of non-HGT genes). The position of eukaryotes, which varies depending on the gene, was not considered in assessing monophyly of eubacteria and archaebacteria.

The 32 remaining proteins were concatenated for analysis. The α parameters used during the tree building process were estimated with the program PamL (JTT+gamma model) [93]. From the concatenation, trees were constructed with ME, Maximum Likelihood (ML) [94] and Bayesian [95] methods. The phylogenies obtained with ME, ML and Bayesian were similar, differing only at non-significant nodes assessed by the bootstrap method [96], with one only significant exception on the position of *M. kandleri *in the Bayesian phylogeny. The sequence alignments and other supplementary data are presented elsewhere [90].

#### Time estimation

Time estimation was conducted separately within each domain (Archaebacteria and Eubacteria) using reciprocal rooting and several calibration points. All time estimates were calculated with a Bayesian local clock approach [97] utilizing concatenated data sets of multiple proteins and a JTT+gamma model of substitution [19,98,99]. The following settings were used: numsamp (10,000), burnin (100,000), and sampfreq (100). This method permitted rates to vary on different branches, which was necessary given the known rate variation among prokaryote and eukaryote nuclear protein sequences [30,44]. Calibration of rate in this method was implemented by assigning constraints to nodes in the phylogeny. Five different initial settings (prior distributions) were used in each domain [see Additional file 4]. These were chosen at intervals of 0.5 Ga starting from 4.5 Ga, which is approximately the age of the Earth and Solar System, to 2.5 Ga, which is slightly before the major rise in oxygen (Great Oxidation Event; GOE) as recorded in the geologic record [32] and related to the presence of oxygenic cyanobacteria. Those constraints pertained to the ingroup root, or deepest divergence in the tree excluding the outgroup. Because of the relatively small number of duplicate genes available for rooting the tree of life, we were unable to estimate the time of the last common ancestor (the divergence of eubacteria and archaebacteria).

For the archaebacterial data set, we included eukaryotes for calibration purposes because reliable calibration points were unavailable among those prokaryotes. In doing so, only proteins in which eukaryotes clustered with archaebacteria were included [30]. An outgroup was used that consisted of representatives of the major groups of eubacteria [90]. We used the fossil and molecular times (separately) of the plant-animal divergence as calibration points, for comparison. The fossil calibration was the first appearance of a representative of the plant lineage (red algae) at 1.198 ± 0.022 Ga [100]. The molecular time estimate for this divergence was 1.609 ± 0.060 Ga from a study of 143 rate-constant proteins [98]. We used the minimum and maximum bounds for these calibration times as constraints in the Bayesian analysis. Although the results of these two different calibrations are provided for comparison, our preferred calibration is the 1.2 Ga fossil calibration because it has the best justification (supporting evidence). Therefore, our summary time estimates for archaebacteria, presented in the timetree (Fig. 3), use only this fossil calibration.

For the eubacterial data set, we used four internal time constraints in separate analyses, all involving the origin of cyanobacteria. The first and most conservative constraint was a fixed origin (minimum and maximum bounds) at 2.3 Ga, which corresponds to the GOE. For the second constraint we used 2.3 Ga as a minimum bound, with no maximum bound. For the third constraint we used a previous molecular time estimate (2.56 Ga) for the divergence of cyanobacteria from closest living relatives among eubacteria, and fixed the minimum (2.04 Ga) and maximum (3.08 Ga) values to the 95% confidence limits of that time estimate [30]. The fourth constraint for the origin of cyanobacteria was set at 2.7 Ga (minimum constraint) based on biomarker evidence for the presence of 2α-methylhopanes [86]. We did not consider the fossil record of cyanobacteria because the earliest indisputable fossils [52] are younger (2000 Ma) than the indirect evidence (GOE) for the presence of these oxygen-producing organisms. Older fossils of cyanobacteria are known but are disputed [52,101]. The use of these four alternative constraints for the origin of cyanobacteria considers most of the widely discussed hypotheses but does not rule out an origin prior to 2.7 Ga. Although the results of the four different calibrations are provided for comparison, our preferred calibration is the 2.3 (minimum) geologic calibration because it has the best justification (supporting evidence). Therefore, our summary time estimates for eubacteria, presented in the timetree (Fig. 3), use only this geologic calibration.

For each of these calibration points, all five initial settings were applied, resulting in 15 and 20 analyses for the Archaebacteria and Eubacteria (respectively). The effects of the different initial settings on the analyses were found to be minimal. A 44% difference in the priors, in fact, generated a maximum 2.7% (average of all significant nodes) difference in the time estimates (fossil calibration point) in the archaebacteria and a maximum 3.5% (average of all significant nodes) difference in the eubacteria (molecular calibration point) [see Additional file 5].

## Authors' contributions

AF assembled and aligned the dataset and conducted initial analyses. FUB conducted phylogenetic and molecular clock analyses and co-drafted the manuscript. SBH directed the research and co-drafting the manuscript.

## Supplementary Material

Additional File 1 Complete time estimation analyses. Estimated times for each node and calibration for Eubacteria and Archaebacteria. The node numbers refer to additional files 1 (eubacteria) and 2 (archaebacteria). Click here for file

Additional File 2 Eubacteria tree. Phylogenetic tree of eubacteria (ME; α = 0.94). Node numbers assigned during the time estimation analyses are represented in italics. Click here for file

Additional File 3 Archaebacteria tree. Phylogenetic tree of archaebacteria (ME; α = 1.20). Node numbers assigned during the time estimation analyses are represented in italics. Click here for file

Additional File 4 Prior distribution values. Mean of the prior distribution for the rate of molecular evolution of the ingroup root node (rtrate) in Eubacteria and Archaebacteria. Click here for file

Additional File 5 Percentage difference. Divergence time estimates and percentage difference due to different ingroup root constraints used under each calibration point. Node numbers refer to additional file 2 (eubacteria) and additional file 3 (archaebacteria). Click here for file
