# Supplementary figures and images for: A genomic timescale of prokaryote evolution: insights into the origin of methanogenesis, phototrophy, and the colonization of land
Source: BMC Evol Biol. 2004 Nov 9;4:44. doi: 10.1186/1471-2148-4-44 (PMC533871; doi:10.1186/1471-2148-4-44)

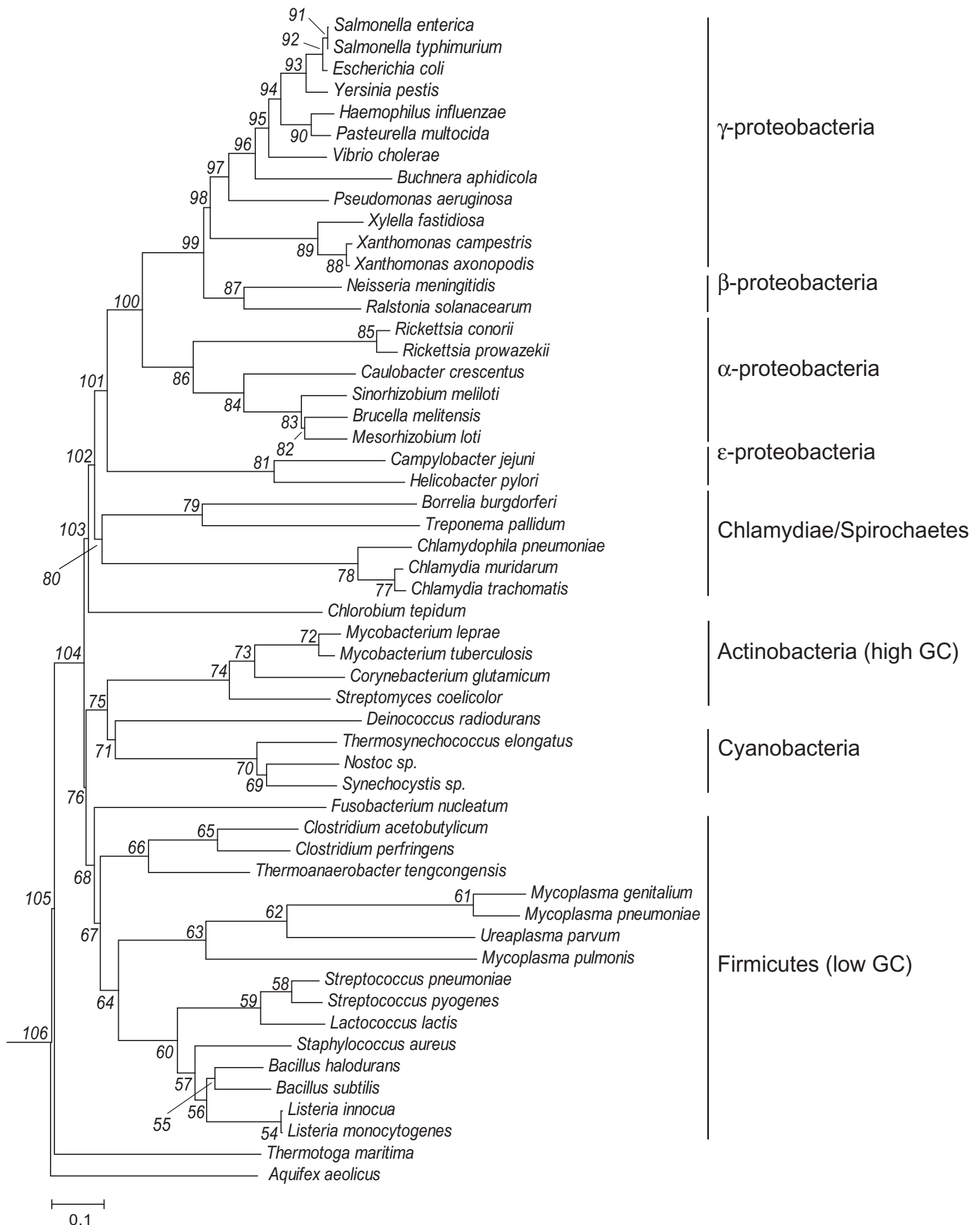

Supplement: Additional File 2 — Eubacteria tree. Phylogenetic tree of eubacteria (ME; α = 0.94). Node numbers assigned during the time estimation analyses are represented in italics. [file 1471-2148-4-44-S2.pdf]

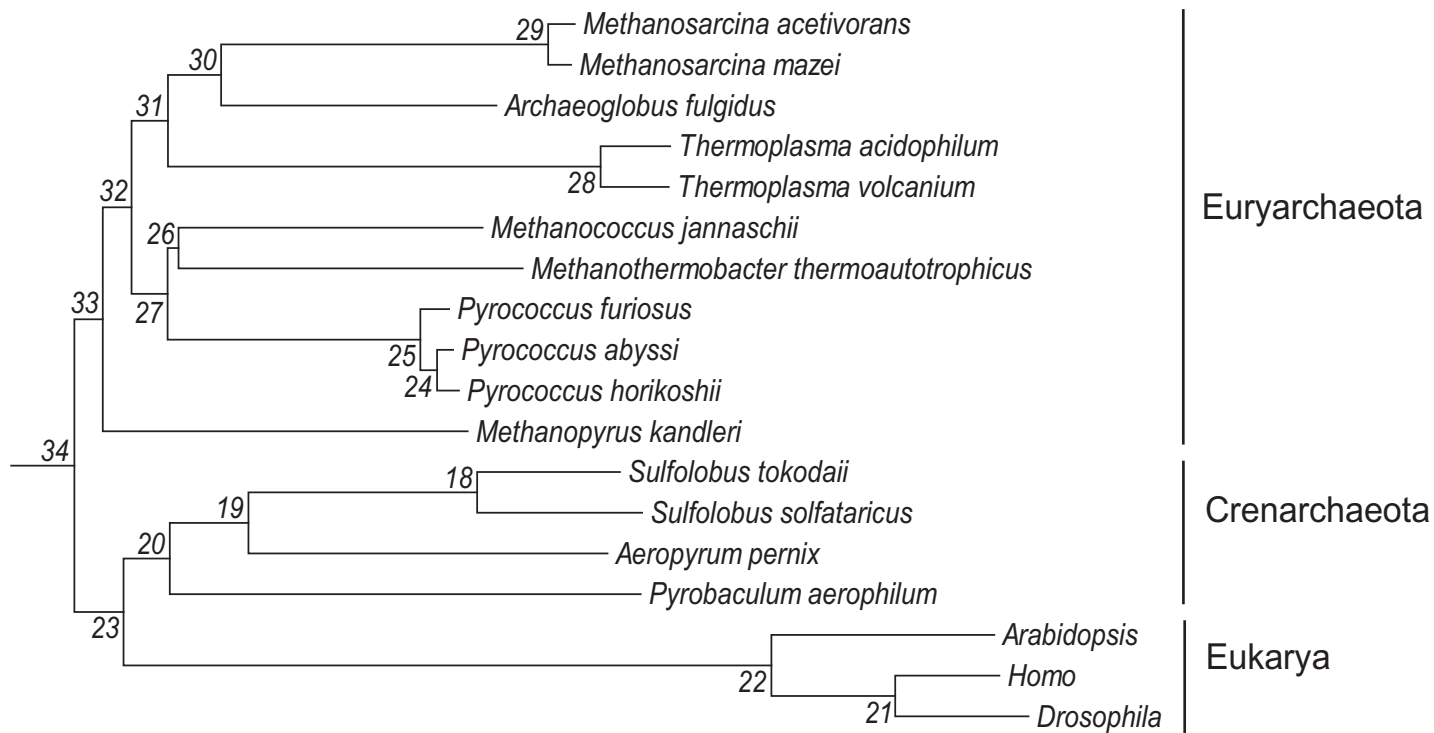

Supplement: Additional File 3 — Archaebacteria tree. Phylogenetic tree of archaebacteria (ME; α = 1.20). Node numbers assigned during the time estimation analyses are represented in italics. [file 1471-2148-4-44-S3.pdf]
