# Supplementary material for: A genomic timescale of prokaryote evolution: insights into the origin of methanogenesis, phototrophy, and the colonization of land
Source: BMC Evol Biol. 2004 Nov 9;4:44. doi: 10.1186/1471-2148-4-44 (PMC533871; doi:10.1186/1471-2148-4-44)
Supplement: Additional File 4 — Prior distribution values. Mean of the prior distribution for the rate of molecular evolution of the ingroup root node (rtrate) in Eubacteria and Archaebacteria. [file 1471-2148-4-44-S4.doc]

**Prior distribution for the rate of molecular evolution**

| EUBACTERIA | |
| --- | --- |
| **Rttm**  (ingroup root constraint) | **Rtrate** |
| 2500 Ma | 0.034 |
| 3000 Ma | 0.028 |
| 3500 Ma | 0.024 |
| 4000 Ma | 0.020 |
| 4500 Ma | 0.019 |

| ARCHAEBACTERIA | |
| --- | --- |
| **Rttm**  (ingroup root constraint) | **Rtrate** |
| 2500 Ma | 0.026 |
| 3000 Ma | 0.022 |
| 3500 Ma | 0.019 |
| 4000 Ma | 0.016 |
| 4500 Ma | 0.014 |
