# Supplementary material for: A genomic timescale of prokaryote evolution: insights into the origin of methanogenesis, phototrophy, and the colonization of land
Source: BMC Evol Biol. 2004 Nov 9;4:44. doi: 10.1186/1471-2148-4-44 (PMC533871; doi:10.1186/1471-2148-4-44)
Supplement: Additional File 5 — Percentage difference. Divergence time estimates and percentage difference due to different ingroup root constraints used under each calibration point. Node numbers refer to additional file 2 (eubacteria) and additional file 3 (archaebacteria). [file 1471-2148-4-44-S5.doc]

**Percentage difference**

| **EUBACTERIA** | | | | | | | |
| --- | --- | --- | --- | --- | --- | --- | --- |
|  |  | **Node 68** | **Node 70** | **Node 71** | **Node 75** | **Node 76** | **Node 92** |
| ***Calibration (Ma)*** | ***rttm (Ma)*** | ***Time (Ma)*** | ***Time (Ma)*** | ***Time (Ma)*** | ***Time (Ma)*** | ***Time (Ma)*** | ***Time (Ma)*** |
| 2300 | 2500 | 2642 | 916 | 2300 | 2511 | 2764 | 91 |
|  | 3000 | 2665 | 888 | 2300 | 2525 | 2792 | 93 |
|  | 3500 | 2663 | 893 | 2300 | 2523 | 2790 | 92 |
|  | 4000 | 2657 | 909 | 2300 | 2519 | 2782 | 92 |
|  | 4500 | 2649 | 915 | 2300 | 2515 | 2776 | 92 |
| Difference | 44.0% | 0.6% | 3.1% | 0.0% | 0.5% | 1.0% | 2.2% |
| 2040-3080 | 2500 | 2669 | 939 | 2328 | 2538 | 2791 | 94 |
|  | 3000 | 2705 | 955 | 2361 | 2574 | 2828 | 94 |
|  | 3500 | 2736 | 952 | 2383 | 2601 | 2862 | 95 |
|  | 4000 | 2762 | 962 | 2407 | 2627 | 2888 | 97 |
|  | 4500 | 2774 | 965 | 2414 | 2636 | 2902 | 95 |
| Difference | 44.0% | 3.8% | 2.7% | 3.6% | 2.4% | 3.8% | 3.1% |
| 2300 min | 2500 | 2895 | 1021 | 2531 | 2757 | 3024 | 100 |
|  | 3000 | 2908 | 1061 | 2556 | 2774 | 3037 | 102 |
|  | 3500 | 2917 | 1028 | 2550 | 2778 | 3050 | 101 |
|  | 4000 | 2942 | 1040 | 2570 | 2801 | 3074 | 103 |
|  | 4500 | 2953 | 1043 | 2581 | 2812 | 3085 | 106 |
| Difference | 44.0% | 2.0% | 3.8% | 1.9% | 2.0% | 2.0% | 5.7% |
| 2700 min | 2500 | 3205 | 1183 | 2835 | 3068 | 3339 | 119 |
|  | 3000 | 3211 | 1193 | 2841 | 3073 | 3344 | 120 |
|  | 3500 | 3214 | 1179 | 2838 | 3073 | 3347 | 119 |
|  | 4000 | 3216 | 1191 | 2844 | 3077 | 3350 | 115 |
|  | 4500 | 3222 | 1176 | 2843 | 3080 | 3357 | 118 |
| Difference | 44.0% | 0.5% | 1.4% | 0.3% | 0.4% | 0.5% | 4.2% |
|  |  | **Node 100** | **Node 101** | **Node 104** | **Node 105** | **Node 106** |  |
| ***Calibration (Ma)*** | ***rttm (Ma)*** | ***Time (Ma)*** | ***Time (Ma)*** | ***Time (Ma)*** | ***Time (Ma)*** | ***Time (Ma)*** |  |
| 2300 | 2500 | 2262 | 2530 | 2889 | 3319 | 3639 |  |
|  | 3000 | 2294 | 2561 | 2921 | 3362 | 3690 |  |
|  | 3500 | 2289 | 2558 | 2920 | 3363 | 3696 |  |
|  | 4000 | 2281 | 2548 | 2912 | 3361 | 3709 |  |
|  | 4500 | 2272 | 2541 | 2905 | 3353 | 3699 |  |
| Difference | 44.0% | 1.4% | 1.2% | 1.1% | 1,3% | 1.9% |  |
| 2040-3080 | 2500 | 2298 | 2562 | 2915 | 3339 | 3652 |  |
|  | 3000 | 2321 | 2592 | 2953 | 3385 | 3705 |  |
|  | 3500 | 2351 | 2623 | 2990 | 3431 | 3758 |  |
|  | 4000 | 2372 | 2648 | 3017 | 3462 | 3794 |  |
|  | 4500 | 2379 | 2660 | 3033 | 3482 | 3817 |  |
| Difference | 44.0% | 3.4% | 3.7% | 3.9% | 4.1% | 4.3% |  |
| 2300 min | 2500 | 2482 | 2772 | 3154 | 3604 | 3931 |  |
|  | 3000 | 2493 | 2785 | 3167 | 3619 | 3945 |  |
|  | 3500 | 2490 | 2788 | 3185 | 3648 | 3983 |  |
|  | 4000 | 2527 | 2819 | 3208 | 3671 | 4010 |  |
|  | 4500 | 2546 | 2834 | 3218 | 3679 | 4016 |  |
| Difference | 44.0% | 2.5% | 2.2% | 2.0% | 2.0% | 2.1% |  |
| 2700 min | 2500 | 2763 | 3070 | 3471 | 3932 | 4246 |  |
|  | 3000 | 2774 | 3078 | 3477 | 3937 | 4250 |  |
|  | 3500 | 2776 | 3080 | 3480 | 3942 | 4256 |  |
|  | 4000 | 2765 | 3078 | 3483 | 3948 | 4267 |  |
|  | 4500 | 2778 | 3087 | 3491 | 3957 | 4274 |  |
| Difference | 44.0% | 0.5% | 0.5% | 0.5% | 0.6% | 0.7% |  |

| **ARCHAEBACTERIA** | | | | | | |
| --- | --- | --- | --- | --- | --- | --- |
|  |  | **Node 29** | **Node 31** | **Node 32** | **Node 33** | **Node 34** |
| ***Calibration (mya)*** | ***rttm (mya)*** | ***Time*** | ***Time*** | ***Time*** | ***Time*** | ***Time*** |
| 1609 | 2500 | 273 | 3169 | 3626 | 3833 | 4149 |
|  | 3000 | 283 | 3182 | 3637 | 3846 | 4164 |
|  | 3500 | 275 | 3135 | 3590 | 3798 | 4117 |
|  | 4000 | 281 | 3191 | 3650 | 3860 | 4182 |
|  | 4500 | 277 | 3179 | 3642 | 3855 | 4182 |
| Difference | 44.0% | 3.5% | 1.8% | 1.6% | 1.6% | 1.6% |
| 1489-1729 | 2500 | 277 | 3166 | 3607 | 3808 | 4119 |
|  | 3000 | 285 | 3195 | 3637 | 3840 | 4153 |
|  | 3500 | 284 | 3211 | 3655 | 3860 | 4175 |
|  | 4000 | 280 | 3214 | 3677 | 3886 | 4206 |
|  | 4500 | 284 | 3243 | 3696 | 3904 | 4223 |
| Difference | 44.0% | 2.8% | 2.4% | 2.4% | 2.5% | 2.5% |
| 1174-1222 | 2500 | 231 | 3039 | 3510 | 3720 | 4042 |
|  | 3000 | 233 | 3051 | 3525 | 3736 | 4061 |
|  | 3500 | 232 | 3093 | 3575 | 3791 | 4123 |
|  | 4000 | 235 | 3118 | 3604 | 3822 | 4158 |
|  | 4500 | 232 | 3124 | 3618 | 3838 | 4177 |
| Difference | 44.0% | 1.7% | 2.7% | 3.0% | 3.1% | 3.2% |
